# Learning machine approach reveals microbial signatures of diet and sex in dog

**DOI:** 10.1371/journal.pone.0237874

**Published:** 2020-08-17

**Authors:** Elisa Scarsella, Bruno Stefanon, Michela Cintio, Danilo Licastro, Sandy Sgorlon, Simeone Dal Monego, Misa Sandri

**Affiliations:** 1 Department of AgroFood, Environmental and Animal Sciences, University of Udine, Udine, Italy; 2 ARGO Open Lab Platform for Genome sequencing, AREA Science Park, Padriciano, Trieste, Italy; University of Illinois, UNITED STATES

## Abstract

The characterization of the microbial population of many niches of the organism, as the gastrointestinal tract, is now possible thanks to the use of high-throughput DNA sequencing technique. Several studies in the companion animals field already investigated faecal microbiome in healthy or affected subjects, although the methodologies used in the different laboratories and the limited number of animals recruited in each experiment does not allow a straight comparison among published results. In the present study, we report data collected from several in house researches carried out in healthy dogs, with the aim to seek for a variability of microbial taxa in the faeces, caused by factors such as diet and sex. The database contains 340 samples from 132 dogs, collected serially during dietary intervention studies. The procedure of samples collection, storage, DNA extraction and sequencing, bioinformatic and statistical analysis followed a standardized pipeline. Microbial profiles of faecal samples have been analyzed applying dimensional reduction discriminant analysis followed by random forest analysis to the relative abundances of genera in the feces as variables. The results supported the responsiveness of microbiota at a genera taxonomic level to dietary factor and allowed to cluster dogs according this factor with high accuracy. Also sex factor clustered dogs, with castrated males and spayed females forming a separated group in comparison to intact dogs, strengthening the hypothesis of a bidirectional interaction between microbiota and endocrine status of the host. The findings of the present analysis are promising for a better comprehension of the mechanisms that regulate the connection of the microorganisms living the gastrointestinal tract with the diet and the host. This preliminary study deserves further investigation for the identification of the factors affecting faecal microbiome in dogs.

## Introduction

A growing number of researches investigated the composition and the variation of gut microbiome in relation to healthy conditions and environmental factors for companion animals and livestock [1, 2]. The microbiota that composes the gastrointestinal (GI) tract of humans and animals has been indicated to be responsible of very important basic functions contribution of metabolic activities, protection against pathogens, sending signals to the immune system and the direct or not affection of most of the physiologic functions [3].

Several studies, both using bacterial culture or molecular methods, aimed to demonstrate that the abundance and the biodiversity of the microbiota increase along the tract [4]. Moreover, the advent of innovative technologies allowed a more frequent utilization of molecular methods to identify the non-culturable bacteria within the canine GI tract. It is estimated that the total microbial load is about 10 times the number of cells present in the host [5].

Dietary intervention studies with clinically healthy dogs have underpinned a high individual variability, which reduced the possibility to find modifications of faecal microbiome in relation to the experimental factor. Moreover, the methodology and the techniques applied in these studies largely vary, limiting the comparison of data obtained from different researches. Upstream methodological issues are the sampling and the DNA isolation procedures using internal protocols or commercial kits, which can affect the yield and purity of the DNA, the integrity and the presence of inhibitors of PCR and lead to different results [6]. The sequencing platforms, the selection of the amplification regions, the depth of sequencing are other upstream choices that affect the final results. According to Allali et al. (2017)[7], the results obtained by 3 sequencing platforms are different in terms of diversity and abundance, even though lead to the comparable biological considerations. Downstream to sequencing, the use of different bioinformatics pipelines [8] is another methodological factor affecting the results of microbial communities. The Human Microbiome Project, launched at the National Institute of Health, aims at the characterization of the microbiome in healthy human subjects in 5 major body sites, namely gut, nasal passage, oral cavity, skin, urogenital tract [9]. In the web site (https://www.hmpdacc.org), the common repository for diverse human microbiome datasets, a minimum reporting standards is implemented. Specifically, the 16S rRNA DNA barcode technique aims at investigating whether there is a core healthy bacterial microbiota in these sites. For livestock and companion animals, minimum standards for sample collections and processing are not agreed yet.

Even though a high variability of microbial composition among studies is found, there are some key bacterial species consistently present in fecal samples of healthy subjects, regardless of the method used, suggesting the presence of a core faecal bacterial community [10]. Nevertheless, the growing knowledge in this topic denotes the existence of a strong variability between microbiome profiles of individuals [11], that has to be taken into consideration when a comparison between several experiments with a limited number of dogs is reported.

Here, we report on an analysis of a dataset of faecal microbiome which supplied 340 bacterial profiles of healthy dogs. These studies were performed following the same protocol, starting from reagents to sequencing platforms and bioinformatics pipeline, to limit the variability associated with the methodology and the technology. Microbial profiles of faecal samples were classified for the factors diets and sex, applying dimensional reduction discriminant analysis followed by random forest analysis to the relative abundances of genera in the faeces.

## Material and methods

### Sample population

The dataset is composed of individual records of dogs obtained from 8 dietary intervention studies (DIS) conducted in the past 5 years, for a total of 340 samples. All the dogs were recruited with the same inclusion criteria, which consisted of healthy conditions, as ascertain by a clinical examination, freedom from external and internal parasites, no pharmacological treatments since at least 3 months. A summary of the studies is reported in the S1 Table. Briefly, dogs were recruited from different living environment for every DIS and they were undergone through diet modulation. The DIS 1 [12], 2 [13], 3 and 8 [14] were private kennels, DIS 4 [15] and 7 were shelters and DIS 5 and 6 were dogs privately owned. For every DIS, the first faecal sample (T0) was collected from dogs that were fed the usual diet they received since at least 6 months (control diet, CTR). Starting from day 1 (T1), dogs were divided in groups and the diet has been changed, as reported in the S1 Table. According to the experimental design, a second faecal sample was collected after 14 (T14) and 28 days (T28) for seven of the eight DIS; only in DIS 6 the second faecal sample was collected after 45 days from the beginning of the study.

The diets involved in the database were grouped in four categories, namely commercial extruded complete diet (K), commercial moist complete diet (W), home-made diet (H) and a raw meat diet with the addition of a complementary food, from here on called Base (B) (www.nutrigenefood.com). A detailed description of the nutritive value of the diets for each experiment is reported in the S2 Table. For the variable sex, the dogs were grouped in males (M), castrated males (MC), females (F) and spayed females (FC). The age and the breed of the dogs were also imputed in the database.

Stool samples were collected with the same protocol for every DIS, from the ground soon after the evacuation, using sterile gloves, introduced into a sterile plastic bag, immediately frozen at -20°C and delivered to the laboratory for storage at -20°C until analysis. Time elapsed from the sample collection to DNA extraction was lower than 30 days.

### Faecal DNA extraction, sequencing and bioinformatic analysis

The entire procedure, starting from the microbial DNA extraction method and ending with taxonomic annotation with a bioinformatic analysis, was standardized and utilized for all the samples. Frozen stools were cleaned from external contaminations with soil with a sterile blade and successively thawed at room temperature. Total DNA was extracted from 150 mg of faeces using a Faecal DNA MiniPrep kit (Zymo research; Irvine, CA, US), following the manufacturer’s instruction. A ZymoBIOMICS™ Microbial Community Standard (Zymo Research, Irvine, CA, USA) was used to assess the efficiency of the entire pipeline, from DNA extraction method to taxonomic annotation. The mock community contains eight bacterial species: *Pseudomonas aeruginosa* (4.2%), *Escherichia coli* (10.1%), *Salmonella enterica* (10.4%), *Lactobacillus fermentum* (18.4%), *Enterococcus faecalis* (9.9%), *Staphylococcus aureus* (15.5%), *Listeria monocytogenes* (14.1%) and *Bacillus subtilis* (17.4%); expected composition of the mock community was given by the manufacturer.

The total amount of DNA extracted and the quality were measured with a QubitTM 3 Fluorometer (Thermo Scientific; Waltham, MA, USA) and libraries were prepared with the amplification of the V3 and V4 regions from the 16S rRNA gene, adding Indexes for sequencing, using a Nextera DNA Library Prep kit (Illumina; San Diego, CA, USA), following manufacturer’s instruction and primers [16]. The resulting amplicons were sequenced with a MiSeq (Illumina; San Diego, CA, USA) in 2x300 paired-end mode, following the standard procedures, for a depth of 50,000 reads.

Raw sequences (FASTQ) of the 8 DIS were collated and processed using the bioinformatic program called Quantitative Insights Into Microbial Ecology 2 (QIIME 2) [17], and uploaded to NCBI Sequence Read Archive (S1 Table). After demultiplexing, sequenced reads that passed the quality check (Phred score ≥30) were annotated for 16S rRNA against the Greengenes database. Chimeras were also detected and then filtered from the reads and the remaining sequences were clustered into Operational Taxonomic Units (OTUs) by using an open reference approach in QIIME 2.

### Computation and statistical analysis

Annotated OTU were imputed on a spreadsheet together with age, sex, breed and the number of the study to allow and facilitate further statistical analysis. The annotates sequences from each sample and each taxonomic level were normalized to ‰ abundance profiles, already known as Relative Abundance (RA). Within each DIS, taxa with RA lower than 1‰ in more than half of the samples were excluded from the statistical analysis [13]. Beta diversity was evaluated with the phylogeny based on UniFrac [18] distance metric and visualized using Principal Coordinate Analysis (PCoA) plots. Permutational multivariate analysis of variance (PERMANOVA) was performed using UniFrac distances by including diets or sex as one of the factors to assess the differences in community composition. For each of the different taxonomic levels, RAs were initially analysed with descriptive statistics to show their distributions, by means of the graphical appraisal of boxplots, reporting maximum, minimum, first and third quartile, mean and median.

Linear discriminant analysis (LDA) was applied for dimensional reduction of variables with the aim to classify dogs for diets (H, B, K, W) or sex (M, F, MC and FC) classes. The technique applied was class dependent with the step-wise method. After this step, we looked at the discriminant functions, to see which one was not linear, and thus which one described and predicted better the class of each observation with the greatest value. Random Forest (RF) classification was built only with the selected variables from LDA that had a non-linear trend: the prediction performance of the classifier is based on the Out-Of-Bag (OOB) data; these data can also evaluate the variable importance, shown with the decrease in prediction rule accuracy by a random permutation of the values in each feature. The ranking parameter mentioned is called Mean Decreased Accuracy (MDA). To confirm what LDA and RF results, a non-parametric Kruskal-Wallis test was applied at the genera level, with Bonferroni correction for multiple comparison. A *p*-value below 0.05 was considered statistically significant [19].

## Results

The partitioning of the 340 dogs for the categorical variables diets and sex are reported in the contingency table (Table 1). The age was not used for classification purposes, since is not straight to compare the age of dogs behaving to different breeds. For the factor diets, the number of observations for the H group represented 30 samples out of 340 (8.82%), whilst dogs fed with K diet accounted for more than 50% of the samples, followed by W and B diets.

**Table 1:** Diets X sex contingency table reporting the number of observations.

Diets	Sex				
	F	FC	M	MC	Total
**B**	21	7	10	18	56
**H**	1	10	3	16	30
**K**	70	9	37	55	171
**W**	53	2	28	0	83
**Total**	145	28	78	89	**340**

F = whole female; FC = spayed female; M = whole male; MC = castrated male; B = Base diet; H = home-made diet; K = commercial extruded complete diet; W = commercial moist complete diet.

The average crude protein content (%/DM) was 27.2%, 27.6%, 36.3% and 30.1% for K, W, H and B diet respectively (Table 2; S2 Table). Indeed, the average lipid content (%/DM) was more variable between diets, being 15.3%, 20.0%, 27.2% and 22.8% for K, W, H and B diets, respectively. For the variable sex, F was the most populated group (145 dogs) and FC the least represented group (28 samples out of 340, 8.24%). The number of records for M and MC in the database was quite similar (78 males and 89 castrated males).

**Table 2:** Mean chemical composition and energy content of the diets fed to the dogs. Details of the diet of each dietary intervention study are reported in the S2 Table.

Item	Unit	Diet			
		K	W	H	B
**Crude protein**	%/DM	27.2	27.6	36.3	30.1
**Lipids**	%/DM	15.3	20.0	27.2	22.8
**Crude fiber**	%/DM	3.3	1.0	1.9	1.2
**Ash**	%/DM	7.2	4.5	11.0	4.2
**NFE**	%/DM	47.0	47.0	23.7	41.8
**ME**	kcal/kg DM	4124	4530	4589	4667

ME = Metabolizable energy; K = commercial extruded complete diet; W = commercial moist complete diet; H = home-made diet; B = Base diet.

**Fig 1:**
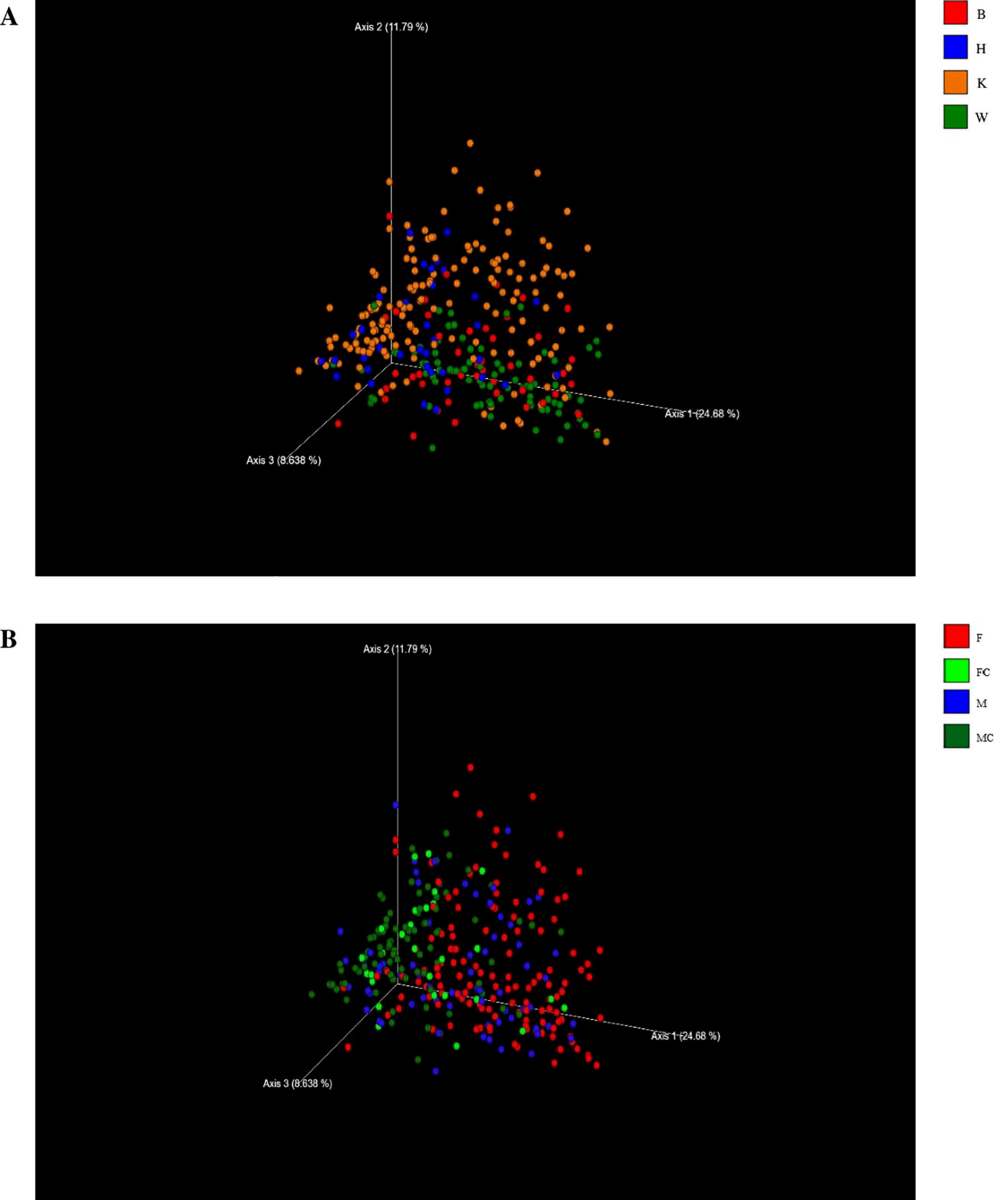
Principal Coordinate Analysis (PCoA) of microbial community from the fecal samples of dogs included in the database; each dot represents a different subject. The 3D PCoA plot was based on weighted UniFrac distances of 16 rRNA gene. (**A**) Subjects are divided in classes based on their different diets. W (green dots) = commercial moist complete diet; K (orange dots): commercial extruded complete diet; H (blue dots) = home-made diet; B (red dots) = Base diet. PERMANOVA confirmed the differences between the four groups of diets for P<0.05. (**B**) Dogs are divided into groups of different sex. F (red dots) = whole females subjects; M (blue dots) = whole males subjects; FC (light green dots) = spayed females subjects; MC (dark green dots) = neutered males subjects. PERMANOVA confirmed the differences between F, M and castrated subjects for P<0.05, but not between FC and MC.

The phylogenetic distance among the samples was investigated using the PCoA of the UniFrac distances of the OTU and in Fig 1A the beta diversity for the factors diets and sex was reported. Two separated clusters of samples from dogs fed with K and W diets were shown, whilst the samples of the H and B groups were more scattered and tended to overlap. The beta diversity with UniFrac distances was also calculated for the factor sex and plotted as PCoA (Fig 1B). Castrated dogs, MC and FC, clustered closer showed a lower beta diversity in comparison to M and F subjects. To evaluate these differences highlighted with the PCoA, we performed a PERMANOVA with the two variables taking into account–diets and sex. A significant contribution of the diets (P<0.05) and sex (P<0.05) was detected. Regarding the sex variable, no differences were observed between FC and MC groups.

### Diet

**Fig 2:**
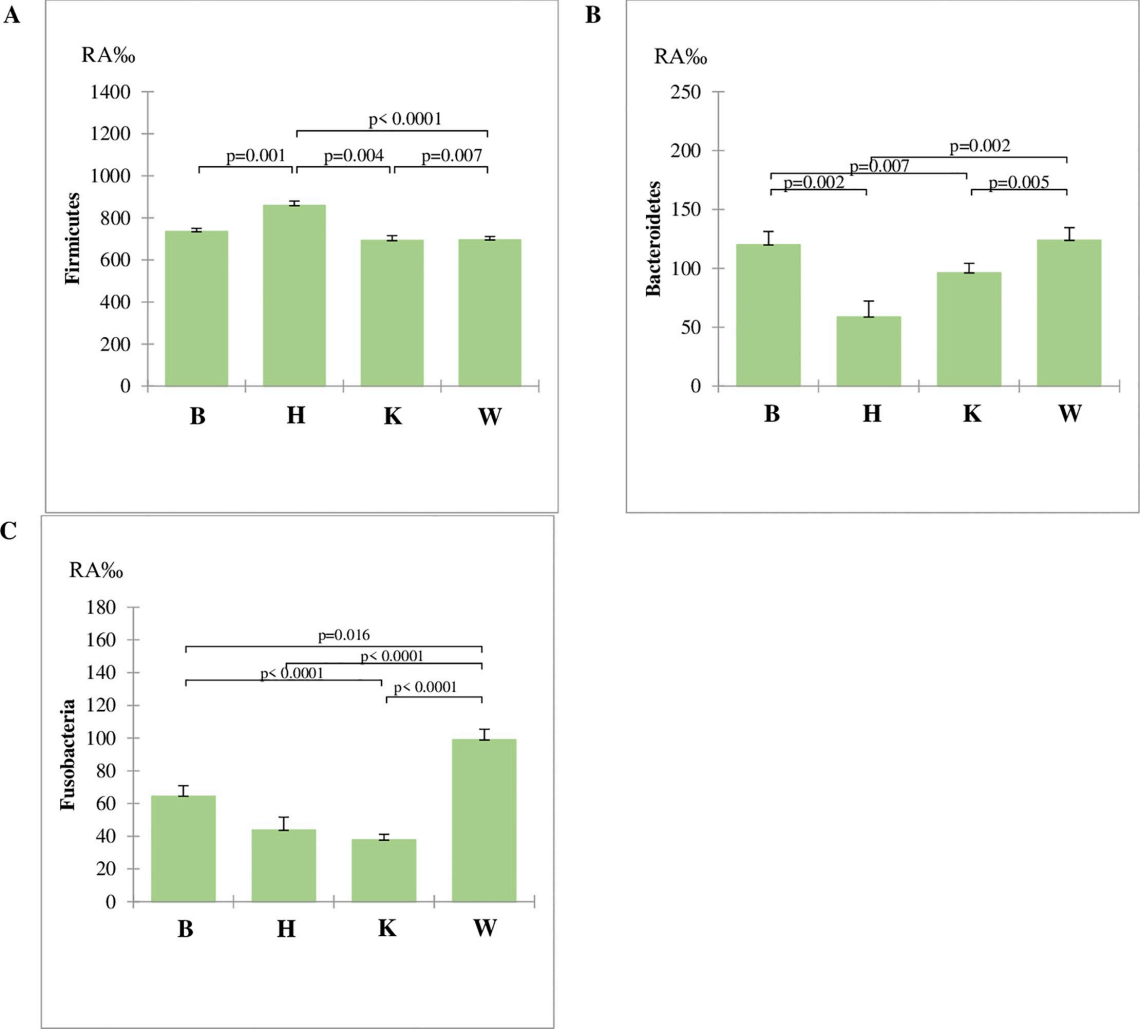
Relative Abundances (RA) for the factor diets of the three represented phyla in the fecal microbiota. RA were compared with the Kruskal-Wallis non-parametric test: (**A**) Firmicutes; (**B**) Bacteroidetes; (**C**) Fusobacteria. Data are reported as mean and standard error. W = Commercial moist complete diet; K = Commercial extruded complete diet; H = Home-made diet; B = Base diet.

Firmicutes were on average the most abundant phylum (mean RA of 713.9 ‰) and a higher mean value (P<0.001) was observed for H diet in comparison to B diet. The mean RA of this latter group was higher (P<0.001) than RAs of K and W diets (Fig 2). The second more abundant phylum was Bacteroidetes (mean RA of 103.4‰), which was highest in B (119.8‰) and W (mean RA 123.6‰) diets in comparison to H and K diets (P<0.001). Significant differences of mean RA of for Bacteroidetes was also observed between H (58.6‰) and K diets (mean RA 96.0 ‰). The RAs of Fusobacteria (mean RA 57.5‰) were higher in W diets in comparison to B diet (P<0.05) and this higher (P<0.001) than H and K diets.

At a genus levels, taxonomic annotations identified 37 features, with *Clostridia* being the most abundant (mean RA 238.5 ‰), having the maximum value (mean RA 334.1 ‰) in H diet and the minimum in K diet (214.1 ‰). Other highly represented bacteria were the *unassigned* genus of Bacteriaceae, *Fusobacterium*, *Lactobacillus*, *Blautia*, *Megamonas*, *Prevotella*, *Ruminococcus*, *Streptococcus* and *Collinsella*. These 37 genera were used in LDA to identify input variables which significantly varied between diets.

**Fig 3:**
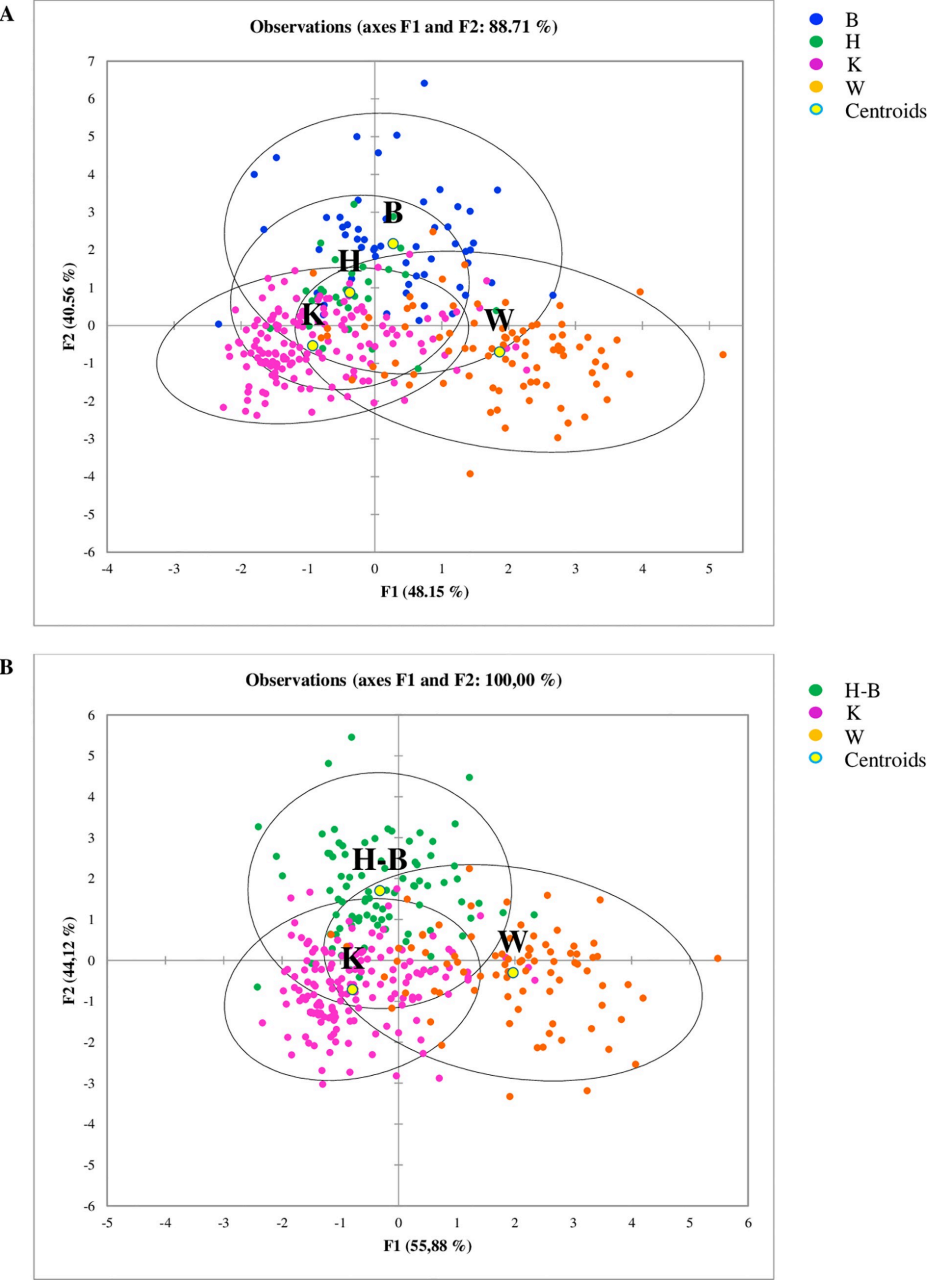
Linear Discriminant Analysis (LDA) based on relative abundances of bacteria genera, showing the clustering of the samples according to the diets. (**A**) result of the LDA using four categories diets, showing that H and B diets are close to each other; (**B**) result of the LDA where H and B diets were collapsed in one group (H-B). W = Commercial moist complete diet; K = Commercial extruded complete diet; H = Home-made diet; B = Base diet; H-B = home-made diet and Base diet collapsed together.

The results of the LDA for the factor diets showed that faecal samples of dogs fed with K and W diets were grouped in two distinct clusters, whilst the samples of dogs fed B and H overlapped, making difficult to separate the two groups (Fig 3A). For the genera *Megamonas*, *Allobaculum*, *Slackia*, *Butyricicoccus*, *Anaerobiospirillum*, *Bacteroides*, *Clostridium*, *Collinsella*, *Escherichia*, *Fusobacterium*, *Oscillospira*, *p-75-a5*, *Peptococcus* and *Roseburia* and for an *unassigned* genus of the family Bacteriaceae, the highest significant coefficients (P<0.0001) were found (S3 Table). Considering the overlapping of the dogs fed B and H diets, the data of these two groups of diets were merged in a new group (named H-B) and the LDA was rerun. The results of the second LDA showed 3 distinct groups (Fig 3B) and, again, for an *unassigned* genus of Bacteriaceae and for the genera *Anaerobiospirillum*, *Bacteroides*, *Clostridium*, *Collinsella*, *Escherichia*, *Fusobacterium*, *Oscillospira*, *p-75-a5* and *Peptococcus* the highest significant coefficients (P<0.0001) were observed. However, significant coefficients were found for other two genera, *Epulopiscium* and *Eubacterium*, and the levels of significance of the coefficients of *Megamonas*, *Allobaculum*, *Slackia* and *Butyricicoccus* were lower than those observed for the LDA with 4 dietary groups.

**Fig 4:**
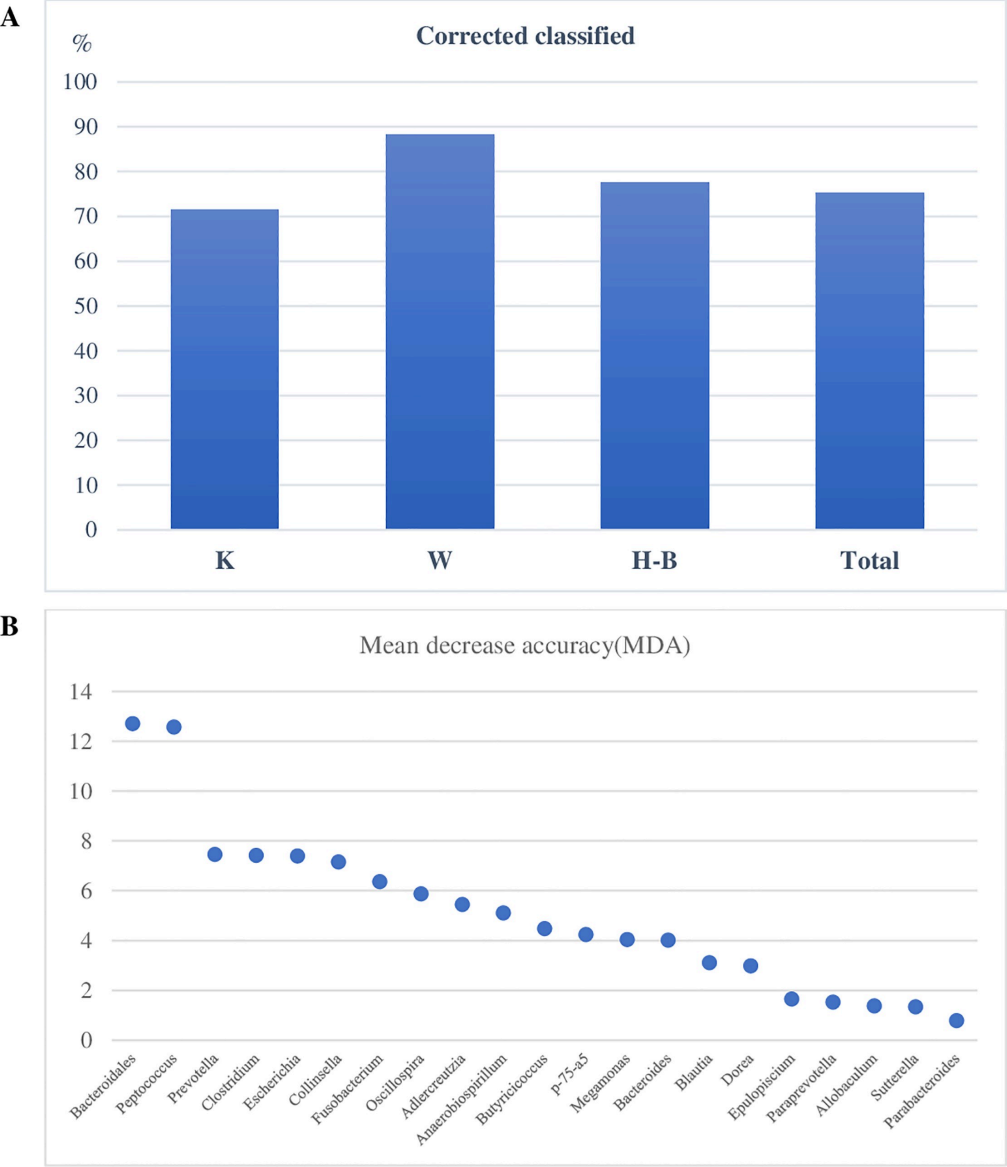
Random Forest (RF) classification of dogs according to the diets based on relative abundances of bacteria genera in the feces. (**A**) percentage of dogs corrected classified based on Out Of Bag data. (**B**) discriminatory power of the genera important for the RF classification of dogs within diets. W = commercial moist complete diet; K = commercial extruded complete diet; H-B = home-made diet and Base diet collapsed together.

A RF classifier was built using the variables having significant linear functions in the LDA for diets. For the factor diets and considering 3 groups (H-B, K, W), the percentage of correctly classified samples was 71.56%, 88.24% and 77.47% for K, W and H-B diets, respectively (Fig 4A; S4 Table). Overall, the RF model correctly classified 75.29% of the total samples. The MDA values indicated the highest discriminatory power of the RF for the *unassigned* genus of Bacteroidaceae and for the genus *Peptococcus* (Fig 4B).

A non -parametric Kruskal-Wallis test was applied to the three groups at the genera level, confirming results of LDA and RF analysis and highlighting some differences between taxa. Results are shown on S5 Table. The main relevant differences were between bacteria that were highly represented in all the three diets: the *unassigned* genus of Bacteriaceae, highly represented in W diet (176.4‰), *Fusobacterium*, having the maximum value in the W diet (98.8‰), *Lactobacillus*, more represented in K diet (66.1‰), *Blautia*, higher in W diet (92.2‰), *Megamonas*, with higher abundance in H-B diet (31.9‰), *Prevotella*, higher in K diet (35.9‰), *Ruminococcus*, more represented in H-B diet (35.3‰), *Streptococcus*, with the maximum value in K diet (71.4‰) and *Collinsella*, higher in H-B diet than in K diet (14.6‰ vs 5.9‰).

### Sex

**Fig 5:**
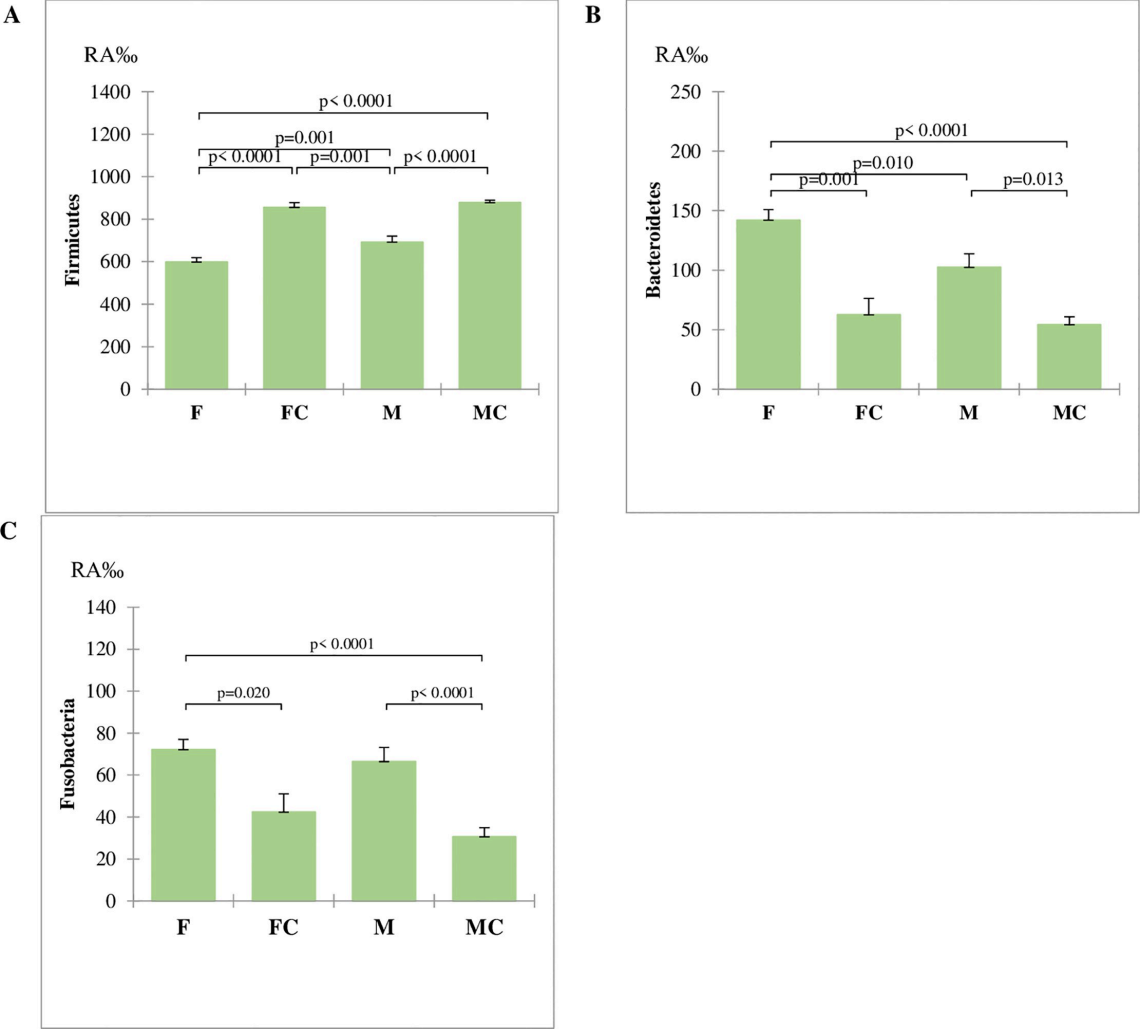
Relative Abundances (RA) for the category sex of the three represented phyla in the fecal microbiota. RA were compared with the Kruskal-Wallis non-parametric test: (**A**) Firmicutes; (**B**) Bacteroidetes; (**C**) Fusobacteria. Data are reported as mean and standard error. F = whole females subjects; M = whole males subjects; FC = spayed females subjects; MC = neutered males subjects.

The most abundant phyla were also affected by sex (Fig 5). The RA of Firmicutes was similar between FC and MC and significantly higher (P<0.001) than F and M dogs. Moreover, the RA of M was higher than that of F (P<0.001). Conversely, the RA of Bacteroidetes was lower in M than in F (P<0.001), but higher (P<0.001) than castrated dogs (FC and MC). Significant differences for the RA of Fusobacterium were observed between entire (F and M) and castrated (FC and MC) dogs.

**Fig 6:**
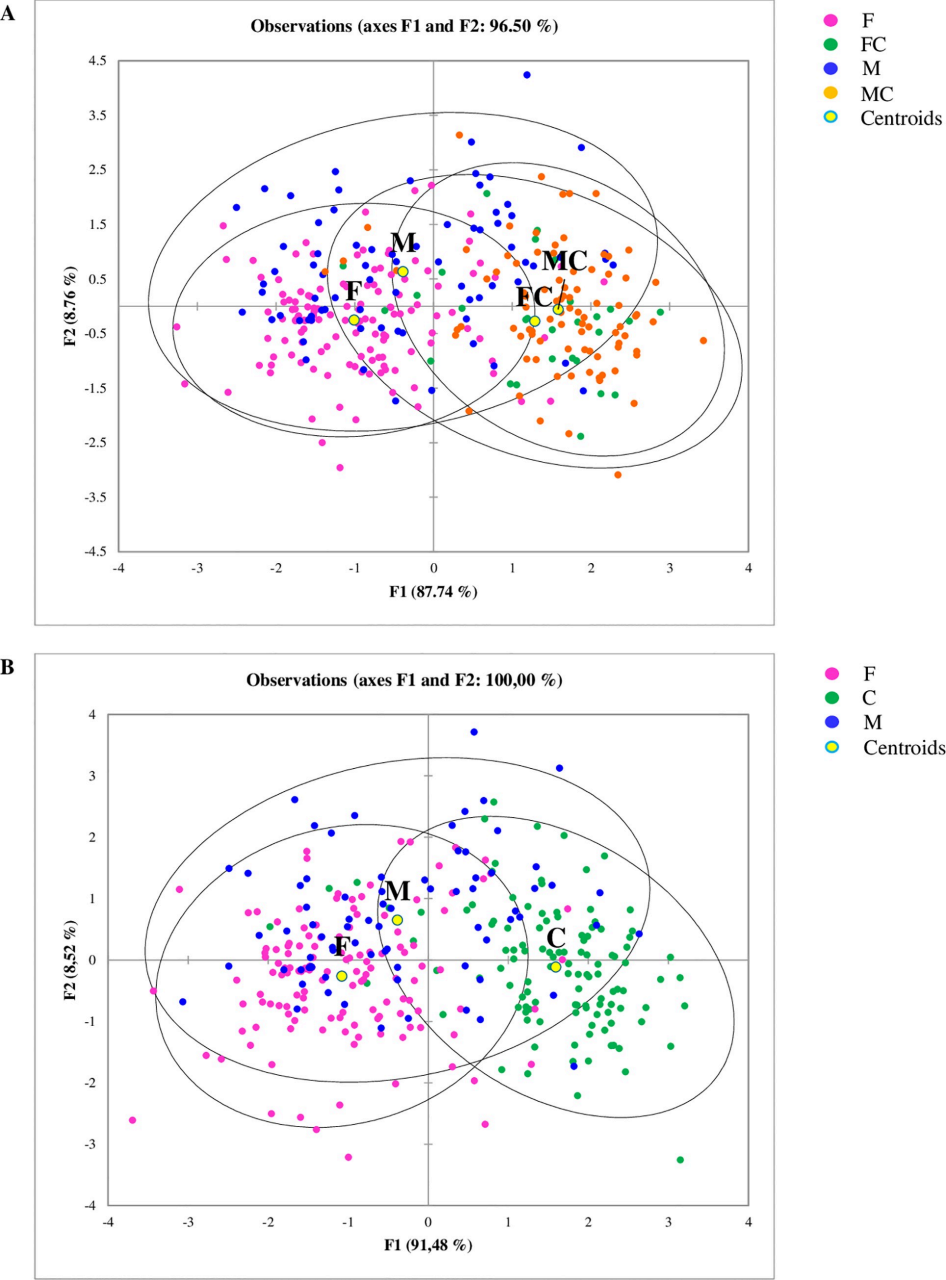
Linear Discriminant Analysis (LDA) based on relative abundances of bacteria genera, showing the clustering of the samples according to the sex. (**A**) result of the LDA using four categories of sex, showing that FC and MC sex are close to each other; (**B**) result of the LDA where FC and MC sex were collapsed in one group. F = whole females subjects; M = whole males subjects; FC = spayed females subjects; MC = neutered males subjects; C = spayed female subjects and neutered male subjects collapsed together.

The LDA using sex as a factor showed that that M and F constituted two distinct groups and separated from to the castrated dogs (MC and FC), which clustered together (Fig 6A). The coefficients for an *unassigned* genus of Bacteriaceae and for the genera *Blautia*, *Dorea*, *Clostridium*, *Fusobacterium*, *Oscillospira*, *Phascolarctobacterium*, *Slackia* and *Streptococcus* were highly significant (P<0.0001, S6 Table). Considering the overlapping of the FC and MC dogs, the data of these two groups were merge in new group (named C) and the LDA was rerun. In the new analysis, significant coefficients were found for the same genera, but other two bacteria resulted highly significant (*Ruminococcus* and *Sutterella*).

**Fig 7:**
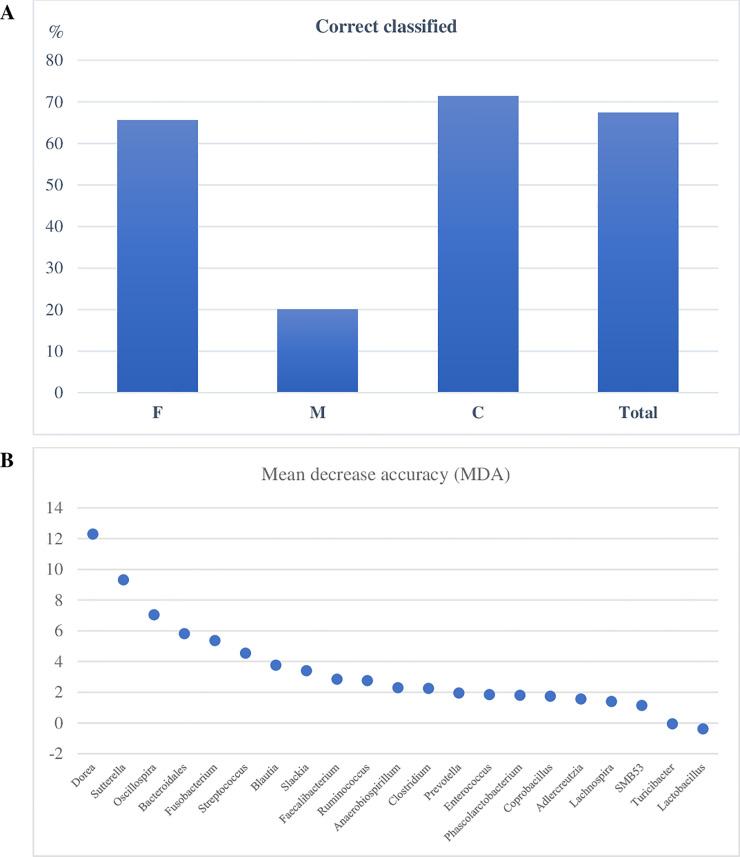
Random Forest (RF) classification of dogs according to the sex based on relative abundances of bacteria genera in the feces. (**A**) percentage of dogs corrected classified based on Out Of Bag data; (**B**) discriminatory power of the genera important for the RF classification of dogs within sex. F = whole females subjects; M = whole males subjects; C = spayed female subjects and neutered male subjects collapsed together.

The RF was used for the classification of data according to the sex factor, with 3 levels (F, M and C), using the variables that had significant linear functions in the LDA. Overall, the percentage of correctly classified total subjects in all categories were 67.35%, meanwhile, and for F and C groups the classification was correct with values of 65.6% and 71.3%, respectively (Fig 7A; S7 Table). The percentages of correctly classified subjects for M was very low. For the sex category, the curve of the MDA of the genera was smoother than for diets, and the higher discriminatory power for the RF classification was shown for *Dorea*, *Sutterella* and *Oscillospira* (Fig 7B).

## Discussion

The PCoAs of Fig 1 indicated that the factors diets and sex had an influence on the beta diversity of gut microbiome, even though a large individual variability was observed. Several studies have pointed out the high variations of faecal microbial communities in healthy dogs [13], suggesting that gut microbiome can be considered an individual fingerprint [20]. However, is not clear if this depend upon the genetic background of the host (nature) or if the environment in a broad sense (nurture) has a prevailing role in shaping the gut microbiome [21]. Apart from the different methodological approaches among the reported studies, it is likely that several factors other than diet could have affected the RA of the phyla. For humans, geographical or ethnic variations, host genetic, immunity, lifestyle and diet or dietary habits have been reported as factors affecting gut microbiota [21]. Sex [22], genetic performances [23], phase of growth, other than diet [24] have been reported to influence gut microbiota in pigs. In healthy dogs, variations of microbiome with age, from weaning to adulthood, were reported [25, 26], with a stabilization of the core gut microbiota at maturity. Since only adult dogs were recruited in the present study, the factor age was not considered.

### Diet

Human microbiome can be split in enterotypes, meaning that individuals can be clustered on the basis of the abundance of microbial taxa of the gut [27], which correspond to specific functional and metabolic activities. The main microbial phyla in the gut of healthy humans are Firmicutes and Bacteroidetes, and their ratio is important for the classification in enterotypes. Mobeen et al. (2018)[28] report that a value of the ratio higher than 1 is prevailing in Asian Countries, while very low values are observed in Burkina Faso and Malaysia and variable ratios were found for Western Countries. Also in dog, the main phyla of faecal microbiota belong to Firmicutes and Bacteroidetes, but their RAs are not been used yet to identify enterotypes. Although a similarity of microbiota between dogs and humans are reported in relation to inflammatory bowel diseases [29] and to the diet [30], contradictory results may suggest that anatomical and physiological differences among species, and the related differences in nutrients and dietary requirements, have to be taken into account. For instance, according to Turnbaugh (2006) [31], obesity in humans and in mice is associated to an increase of Firmicutes and a reduction of Bacteroidetes; indeed obese dogs have a significant decrease of Firmicutes and increase of Protebacteria [32], whilst Bacteroidetes and Fusobacteria are unchanged. Furthermore, contradictory results in the literature make difficult the identification of a gut microbiome core in dogs. The shift on RA of Bacteroidetes and Firmicutes in relation to the diets (Fig 2) did not correspond with that reported in other researches in dogs. In the study of Mori et al. (2019) [33], a low fat high protein diet caused a reduction of Bacteroidetes and an increase of Fusobacteria. Algya et al. (2018) [34] reported a significant increase of Bacteroidetes and Fusobacteria and a decrease of Firmicutes in raw diet, based on chicken and sweet potatoes, in comparison to kibble diet. Indeed, the increase of Fusobacteria RA in the W diet was in agreement with these authors, but not for Bacteroidetes, which showed a very low RA in the H diet, that contains the highest lipid content (Table 2). Bacteroidetes, Gram negative either aerobic and anaerobic bacteria, originated from ancestors living in environment and possess high ability to utilize different carbohydrates, thanks to a large number of carbohydrate-active enzymes that enable them to ferment dietary glycans either from the diet of from the host [35]. For this reason, Bacteroidetes easily adapt to diverse environmental conditions [36], which determine their RA across the hosts [37, 38].

Modification of gut microbiota in response to dietary treatments have been observed also at other taxonomic levels in healthy dogs. Algya et al (2018) [34] reported an increase of genus *Clostridium* in dogs fed with a commercial extruded diet, in comparison to dogs fed with a raw meat diet and a commercial moist diet. On the contrary, Bermingham et al (2017) [39] observed a decrease of *Clostridium* in dogs fed with a commercial extruded diet in comparison to the raw meat diet. Other variations were observed for *Lactobacillus* and *Dorea*, meanwhile *Prevotella*, *Turicibacter*, *Ruminococcus* and *Megamonas* increased in kibble diet group rather than raw meat diet group. A similar result has been reported by Herstad et al. (2017) [40], where *Clostridium* and *Dorea* increased when dogs were fed with a high content of boiled minced beef compare to the control diet based on a commercial extruded diet. Kim et al (2017) [41] confirmed a lower abundance of the family Clostridiaceae in dogs fed with a commercial extruded diet in comparison to a home made diet. Also in a study of Sandri et al (2017) [12], the RAs of *Clostridium* and *Prevotella* were higher in kibble based diet, but *Lactobacillus* and *Megamonas* decreased in dog fed with a meat based diet. *Megamonas* was also significantly higher in another study [2], where the same comparison was reported. In this study, also the abundances of *Lactococcus*, *Clostridium* and *Fusobacterium* genera increased after the administration of a H diet. Interestingly, *Fusobacterium* showed the highest significant difference in W diet, incomparison to H-B and K diet. Also the Ras of *Prevotella*, *Slackia* and *Collinsella* were similar in W and H-B diets and differed from K diet. At the best of our knowledge, straight comparison on faecal microbiome between a complete moist diet and a complete extruded diet is not reported in literature. These findings require to be confirmed with further studies.

These contradictory results can be due to the variability of the environments in which the studies were carried out, together with the methodological issues previously reported. Moreover, the gut microbiome is an ecosystem that strongly interacts with the host and the variations of the abundances are probably better depicted with a multivariate approach. The prevalence of specific taxa can be effective to identify dysbiosis events, but probably not enough to characterize the conditions of the dogs, as the overall assessment of several, in not all, taxa remains necessary [42]. Vazquez-Baeza et al (2016) [29] reported that the diversity and structure of microbial community, more than the variation of single taxa, could be used as a signature of the faecal microbiota to separate dogs with IBD from healthy dogs.

The relationship between a dependent variable and several independent variables was investigated with LDA, which identified significant features to separate the database according to the diet factor (Fig 4). Interestingly, the data of H and the B diets were merged in a cluster, even though the composition of these two diets was different (Table 2). It is likely that the presence of raw meat and the physical form of these diets had a similar impact on shaping gut microbiome. To classify dog data on the basis of diet, a RF was used, an approach that was successfully applied to create a urban microbial fingerprint based on microbioma [43] and to identify healthy dogs or affected by inflammatory bowel disease using faecal microbial profile [29]. The results showed an excellent classification accuracy (Fig 4) and indicated that some genera are more responsive to dietary factors. A limitation of this classification could be that while 71 dogs changed diet from the T0 sample and the following sampling times, 64 dogs at T14 or T28 received the same diet. For these latter subjects, it would be possible that the two fecal samples from the same dog are very if not completely similar, thus ending up including the same sample twice. However, it should be considered that 19 of these 64 dogs were misclassified by RF, suggesting that other factors rather than diet can shape the faecal microbiome of dogs, also in a relatively short time. Although we are aware that this can be a limitation for the current classification, the aim of this study was to develop a model more than assessing a precise identification of diet responsive and core faecal microbiome in dogs, which indeed deserves further investigations.

### Sex

Very limited information is available for the variation of gut microbiome in relation to sex in dogs. Coelho et al (2018) [30] did not find significant differences in microbiome composition between male and female and the results are partly in agreement with our study. As can be seen in Fig 5, the main differences were found between whole (F and M) and castrated (FC and MC) dogs, while F and M were more similar. These differences were confirmed by the multivariate approach of LDA (Fig 6) and by the learning machine classifier (Fig 7). Sex-dependent effects on the microbiome have been reported in animal models [44, 45]. In mice, male and female microbiota diverge after puberty, reflecting the sex bias in expression of autoimmune diseases, such as type 1 diabetes. Although the mechanism of sexual influence remains unclear, a bidirectional interaction of microbiota with endocrine status of host is likely, considering that the divergence between male and female microbiota can be reversed by male castration [44]. Our findings on the effect of sex on gut microbiome are promising and confirm what already emerged for human and other animal models. Despite this, it is still difficult to draw definitive considerations, as the relationship between intestinal microbiome and sex is still poorly studied and not clear.

The study by Markle et al (2013) [44] shows that there is a well-defined structure of the intestinal microbial community that develops and diversifies during sexual maturation, indicating this process as a determining factor. The results of this study are based on animals raised in the same conditions: our results are obtained through a very standardized sample collection, processing and data analysis pipeline, but the subjects were not in the same environment. Despite this, our results agree with that highlighted by other studies [44, 45], confirming and strengthening the hypothesis that there is a bidirectional interaction between microbiota and hormone levels of the host. In the literature it has also been highlighted how castration can reverse the divergence of the microbiome between males and females [44]. Indeed, although the FC and MC variables have a limited number of subjects, we managed to highlight how much these two variables can actually be superimposed, not discriminating each other; by joining them, we managed to obtain a single class extremely discriminatory towards subjects F and M. Although F and M subjects were underrepresented in H diet and that no MC and only 2 FC dogs were fed with W diet, the strong classification power obtained with RF suggested that sex can play a role in shaping the gut microbiome and thus is a factor that requires to be unraveled with appropriated and deeper investigation.

## Conclusion

In this study, a collection of 16S rRNA data were used to investigate if diet composition and sex affected fecal microbial community in healthy dogs. The association of discriminant analysis with learning machine indicated that diet and sex are factors inferring fecal microbiome and that dogs can be clustered on the basis of them. However, the study has some limitations, due to the underrepresentation of dogs in the H diet and in the entire female and male categories. To generalize these preliminary results, a larger dataset is required with the contribution of the scientific community, which can be possible if a basic standardization of the protocols among laboratories will be agreed.

## Supporting information

S1 Table Summary of the dietary intervention studies that were included in the dataset. Kibble = Complete extruded food; Kibble-P = Complete extruded food + probiotic; Kibble-D0 = complete extruded food without polyphenols; Kibble-D1 = complete extruded food with polyphenols, 1 mg/kg; Kibble-D3 = complete extruded food with polyphenols, 3 mg/kg; Moist-S = complete moist food, with sunflower oil and salmon oil; Moist-H = complete moist food, with hemp oil; Moist-D0 = complete moist food without polyphenols; Moist-D1 = complete moist food with polyphenols, 1 mg/kg; Moist-D3 = complete moist food with polyphenols, 3 mg/kg; Home = home based diet, with raw meat; Base-1 = mixed diet, beef raw meat and complementray vegetable food; Base-2 = mixed diet, beef raw meat and complementray vegetable food; Base-3 = mixed diet, beef raw meat and complementray vegetable food.
(XLSX)

S2 Table Chemical compositions and nutritive values of the diets of the Dietray Intervention Studies (DIS). Kibble = Complete extruded food; Kibble-P = Complete extruded food + probiotic; Kibble-D0 = complete extruded food without polyphenols; Kibble-D1 = complete extruded food with polyphenols, 1 mg/kg; Kibble-D3 = complete extruded food with polyphenols, 3 mg/kg; Moist-S = complete moist food, with sunflower oil and salmon oil; Moist-H = complete moist food, with hemp oil; Moist-D0 = complete moist food without polyphenols; Moist-D1 = complete moist food with polyphenols, 1 mg/kg; Moist-D3 = complete moist food with polyphenols, 3 mg/kg; Home = home based diet, with raw meat; Base-1 = mixed diet, beef raw meat and complementray vegetable food; Base-2 = mixed diet, beef raw meat and complementray vegetable food; Base-3 = mixed diet, beef raw meat and complementray vegetable food.
(XLSX)

S3 Table Comparison of the mean relative abundances (RA) and coefficients of the linear discriminant analysis (LDA) using genera as input variables and considering 4 diets categories (LDA1) or 3 diets categories (LDA2). For this latter, B and H diets were collapsed together. W: Commercial moist complete diet; K: Commercial extruded complete diet; H: Home-made diet; B: Base diet; H-B: home-made diet and Base diet collapsed together.
(XLSX)

S4 Table Confusion matrix of the linear discriminant analysis (LDA), considering 4 diets categories (LDA1) or 3 diets categories (LDA2). **For this latter, H and B diets were collapsed together.** W: Commercial moist complete diet; K: Commercial extruded complete diet; H: Home-made diet; B: Base diet; H-B: home-made diet and Base diet collapsed together.
(XLSX)

S5 Table Comparison of the mean relative abundances (RA) through a non-parametric Kruskal-Wallis test of the three groups of diets. W: Commercial moist complete diet; K: Commercial extruded complete diet; H-B: home-made diet and Base diet collapsed together; a, b and c on the same row denote means which significantly differ for P<0.05.
(XLSX)

S6 Table Comparison of the mean relative abundances (RA) and coefficients of the linear discriminant analysis (LDA) using genera as input variables and considering 4 sex categories (LDA1) or 3 sex categories (LDA2). For this latter, FC and MC sex were collapsed together. F: whole females subjects; M: whole males subjects; FC: spayed females subjects; MC: neutered males subjects; C: spayed females subjects and neutered males subjects collapsed together.
(XLSX)

S7 Table Confusion matrix of the linear discriminant analysis (LDA) considering 4 sex categories (LDA1) or 3 sex categories (LDA2). For this latter, FC and MC were collapsed together. F: whole females subjects; M: whole males subjects; FC: spayed females subjects; MC: neutered males subjects; C: spayed females subjects and neutered males subjects collapsed together.
(XLSX)
